# Remimazolam-based anesthesia with flumazenil allows faster emergence than propofol-based anesthesia in older patients undergoing spinal surgery: A randomized controlled trial

**DOI:** 10.1097/MD.0000000000036081

**Published:** 2023-11-17

**Authors:** Yukari Toyota, Takashi Kondo, Kyoko Oshita, Toshiaki Haraki, Soshi Narasaki, Kenshiro Kido, Satoshi Kamiya, Ryuji Nakamura, Noboru Saeki, Yousuke T. Horikawa, Yasuo M. Tsutsumi

**Affiliations:** a Department of Anesthesiology and Critical Care, Hiroshima University Hospital, Hiroshima, Japan; b Department of Anesthesiology, JA Hiroshima General Hospital, Hiroshima, Japan; c Department of Pediatrics, Sharp Rees-Stealy Medical Group, San Diego, CA.

**Keywords:** emergence, flumazenil, general anesthesia, propofol, remimazolam

## Abstract

**Background:**

Remimazolam is a novel, ultrashort-acting benzodiazepine that can be antagonized by flumazenil. This study aimed to determine whether remimazolam-based anesthesia with flumazenil provides a more rapid emergence than propofol-based anesthesia in older patients undergoing spinal surgery.

**Methods:**

This was a prospective, single-blind, randomized controlled trial. Forty-four patients > 75 years old who had undergone spinal surgery were enrolled in this study. They were randomly assigned to the remimazolam or propofol group (1:1) using a computer randomization system stratified by age and body weight. For anesthesia induction and maintenance, remifentanil was administered at a defined dose in both groups, and remimazolam or propofol was adjusted to maintain the bispectral index or state entropy monitoring within 40–60. All anesthetics were discontinued simultaneously after the postoperative X-ray and 0.5 mg flumazenil was administered to the remimazolam group. The primary outcome was extubation time after discontinuing anesthesia, and the secondary outcomes were time to eye opening, obeying commands, and achieving a white fast-track score (WFTS) of 12.

**Results:**

Thirty-nine patients were finally analyzed: remimazolam group (n = 20), propofol group (n = 19). There were no significant differences in intraoperative variables, such as operative time, anesthesia time, and patient background, between the 2 groups. Extubation times were significantly shorter in the remimazolam group than in the propofol group (4 vs 8 minutes, *P <* .001). The time to eye opening, obeying commands, and achieving a WFTS of 12 were significantly shorter in the remimazolam group (*P <* .001, for all comparisons).

**Conclusion:**

Remimazolam-based anesthesia with flumazenil resulted in a faster emergence than propofol-based anesthesia in older patients undergoing spinal surgery.

## 1. Introduction

Delayed emergence from general anesthesia interferes with operating room (OR) management and increases adverse events such as delayed recovery of airway reflexes.^[1,2]^ Older patients are more prone to delayed emergence because they have poorer organ function, reduced brain function, and delayed metabolism and excretion of medications than younger patients.^[3]^

Spinal surgery, which often involves older patients, requires intraoperative motor-evoked potential (MEP) monitoring to reduce perioperative neurological complications, which limits the use of inhaled anesthetics and muscle relaxants that interfere with MEP monitoring. Therefore, total intravenous anesthesia using propofol is recommended for anesthesia maintenance, and sufficient anesthesia depth must be maintained to achieve immobility without muscle relaxants during spinal surgery.^[4]^

Propofol is primarily metabolized and excreted by the liver and kidneys. Therefore, older patients are likely to have delayed emergence and require smaller doses than younger patients.^[3]^

Remimazolam is a new benzodiazepine that can be administered continuously and antagonized by flumazenil. This new intravenous anesthetic is an ultra-short-acting agent that enables more rapid induction and emergence than the conventional benzodiazepine midazolam. It has a similar structure to midazolam; however, it is rapidly metabolized by tissue esterases, primarily in the liver, and its metabolites are inactive.^[5]^ Therefore, it is expected to provide rapid emergence even when used in older patients.

The purpose of this study was to compare the state of emergence in older patients who underwent spinal surgery under general anesthesia using remimazolam and propofol and to determine whether remimazolam-based anesthesia provides a more rapid emergence than propofol-based anesthesia. Since remimazolam is approved for general anesthesia only in Japan and Korea, and studies on the state of emergence from general anesthesia using this drug are still few, especially limited to older patients undergoing spinal surgery, we believe it is significant to report the results of this study using remimazolam.

## 2. Methods

### 2.1. Study design

This prospective, multicenter, single-blind, randomized controlled study was conducted at Hiroshima University Hospital and JA Hiroshima General Hospital (a regional hospital). The study was approved by the Ethics Committee of Hiroshima University on November 25, 2020 (C-306) and the Ethics Committee of JA Hiroshima General Hospital on February 22 2022 (21–73) and registered in the UMIN Clinical Trials Registry database (UMIN000042568, first registration date 26/11/2020). Written informed consent was obtained from all patients, and all study-related procedures were performed in accordance with the Declaration of Helsinki.

### 2.2. Participants

Patients who met the inclusion criteria underwent spinal surgery including laminoplasty, laminectomy, posterior spinal fusion, and resection of spinal tumors.

The inclusion criteria were as follows: >75 years old, American Society of Anesthesiologists (ASA) physical status classification 1 to 3, and written informed consent. The exclusion criteria were as follows: liver dysfunction (Child-Pugh class B or C), renal replacement therapy, morbidly obese (BMI: body mass index ≥ 35), dementia, atypical surgery, and contraindications for use of anesthetics.

### 2.3. Randomization and masking

The patients underwent single-blind randomization (1:1) into 2 groups (remimazolam group - group R, or propofol group - group P) via the Research Electronic Data Capture (REDCap) computer randomization system stratified by age (75–79 years old or ≥ 80 years old) and body weight (<60 kg or ≥ 60 kg). Patients and operators were blinded to group identification, whereas the attending anesthesiologist could not be blinded to group identification because of the markedly different appearance of the 2 anesthetics.

### 2.4. General anesthesia protocol

All patients were randomly managed by staff anesthesiologists at each hospital. None of the patients received any premedication. Standard monitoring included electrocardiography, noninvasive blood pressure or arterial blood pressure monitoring, pulse oximetry, capnography, electroencephalogram-based monitoring using the bispectral index (BIS, Aspect Medical Systems, Newton, MA) at Hiroshima University Hospital or Spectral Entropy (GE Healthcare, Helsinki, Finland) at JA Hiroshima General Hospital, and neuromuscular monitoring via train-of-four monitoring.

General anesthesia was induced with a continuous intravenous infusion of remifentanil at 0.25 µg/kg/min, followed by continuous infusion of remimazolam at 12 mg/kg/h in group R or target-controlled infusion of propofol with an initial target of 3 µg/mL in group P. Remifentanil doses were calculated based on actual body weight for patients with a BMI < 25 and ideal body weight for patients with BMI ≥ 25. Other anesthetics were calculated based on actual body weight. Rocuronium was administered at 0.6 mg/kg only for intubation. Following anesthesia induction, the ventilator settings were adjusted to maintain an oxygen saturation (SpO_2_) ≥ 95%, and the end-tidal carbon dioxide concentration was in the range of 30-–40 mm Hg. Blood pressure was adjusted to maintain a systolic pressure > 90 mm Hg using fluid therapy or intermittent administration of vasopressors.

In both groups, for anesthesia maintenance, remifentanil was maintained at approximately 0.25 µg/kg/min and tapered to 0.05 µg/kg/min after ropivacaine wound infusion before skin closure. Remimazolam or propofol was adjusted to keep the BIS or state entropy values in the range of 40 to 60 after the patients lost consciousness.^[6]^ The maximum infusion rate of remimazolam for anesthesia maintenance was set at 2 mg/kg/h according to the attached document. Acetaminophen and flurbiprofen were administered during the subcuticular closure. The dose of acetaminophen was 15 mg/kg for patients < 50 kg actual body weight and 1000 mg for patients ≥ 50 kg body weight. Fifty milligrams of flurbiprofen was added to acetaminophen for patients with normal renal function. All anesthetics were discontinued after postoperative radiography in the supine position. In group R, flumazenil (0.5 mg) was administered simultaneously as the discontinuation of anesthetic. The emergence of anesthesia was observed by gently tapping the patient on the shoulder while calling their name every minute after the end of anesthetic administration.

The criteria for extubation are as follows: SpO_2_ ≥ 90% with a fraction of inspiratory oxygen ≤ 40%s, tidal volume ≥ 5 mL/kg, respiratory rate < 30/min, stable circulation, and the ability to respond.^[7]^ When the patient complained of pain after extubation, 50 µg of fentanyl was administered as a rescue analgesic drug.

We measured the following times: from discontinuing administration of anesthetics to eye-opening, obeying commands, extubation, and achieving a white fast-track score (WFTS) of 12. The WFTS was used to evaluate the ability to be discharged from the OR (see Supplementary Table S1 online, http://links.lww.com/MD/K691). After extubation, the patient was allowed to leave the OR after confirming that the WFTS was ≥ 12 and that no adverse events had occurred. Three hours after leaving the room, the presence or absence of adverse events was reevaluated and the evaluation was completed.

### 2.5. Outcome measures

The primary outcome was the elapsed time from the discontinuation of anesthesia to extubation. Extubation time can be an indicator of the emergence of the patient from general anesthesia, as extubation requires the recovery of respiratory function and consciousness.

The secondary outcomes were the elapsed time from the discontinuation of anesthesia to eye opening, obeying commands, and achieving a WFTS of 12.

Other measured outcomes included the duration of surgery and anesthesia, dose of each anesthetic, frequency of post-anesthetic adverse events, and WFTS before leaving the OR.

### 2.6. Sample size

Previously, we found that the average time to extubation at our institution was 17 ± 5 minutes in 37 patients who were > 75 years old and underwent spinal surgery under propofol-remifentanil anesthesia from April 2019 to March 2020 in Hiroshima University Hospital.

The G*power 3.1.0 program (http://www.gpower.hhu.de/) was used to calculate the required sample size. Our sample size calculations for the hypothesis that the remimazolam group makes the time to extubation 30% shorter than that of the propofol group, in reference to similar studies comparing different anesthetics,^[8,9]^ resulted in 19 patients per group.

We used a type 1 error of α = 0.05, and a power of 0.80 to calculate sample sizes.

Considering a dropout rate of 15 %, 22 patients per group were enrolled in this study.

### 2.7. Statistical analyses

All data are expressed as interquartile range, or numbers (%). Continuous variables were compared using the Mann–Whitney U test, and the Fisher exact test was used to compare categorical variables. Statistical significance was set at *P <* .05. All statistical analyses were performed using the PRISM software (version 8.0; GraphPad Software, San Diego, CA).

## 3. Results

**Figure 1.**
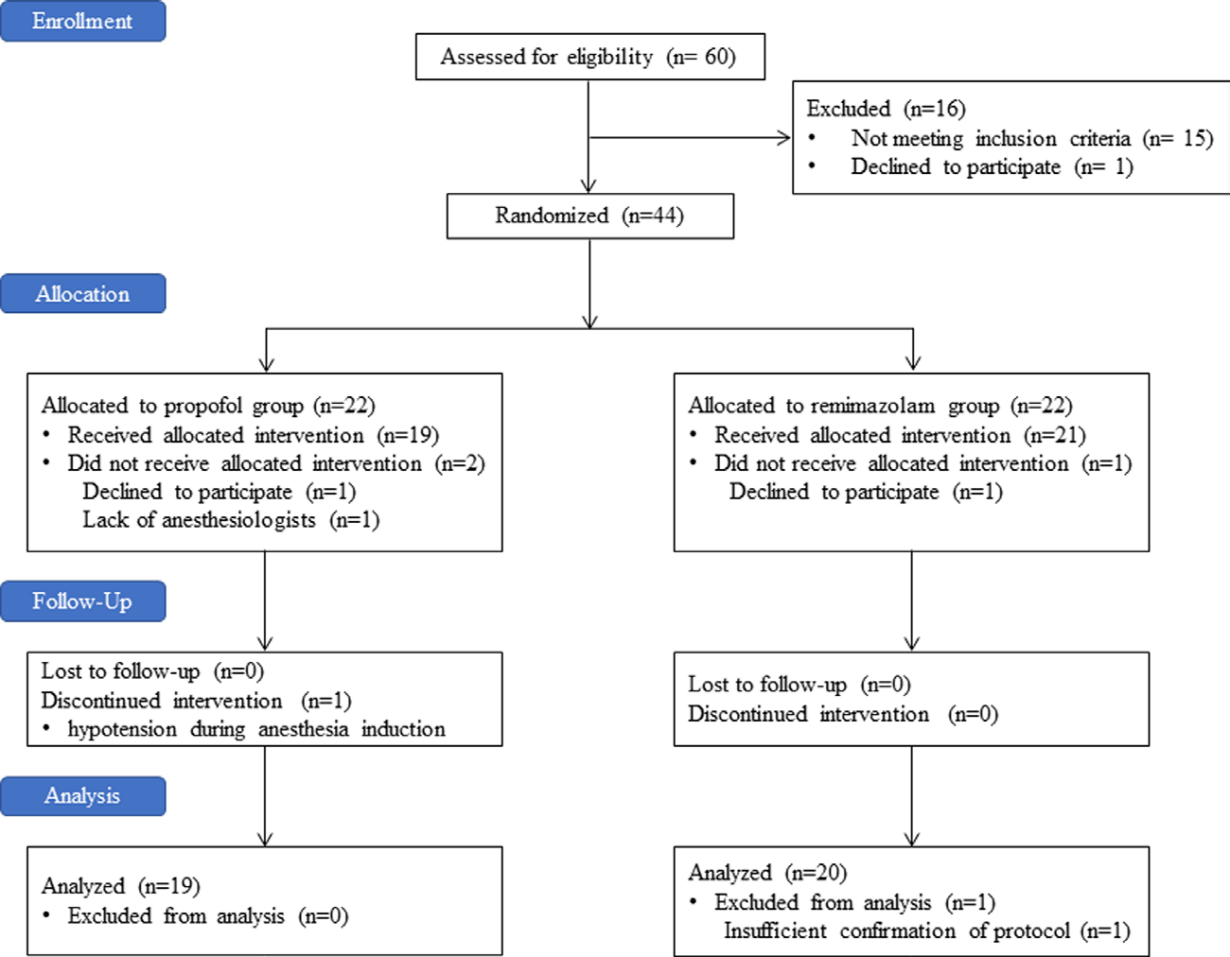
The CONSORT flow diagram.

The CONSORT flow diagram of this study is shown in Figure 1.

Forty-four patients were enrolled between January 2021 and September 2022 and were randomly allocated to either group R (n = 22) or group P (n = 22). Following randomization, 2 patients declined to participate. One patient was unable to receive the allocated intervention because there was no anesthesiologist available to conduct this study because of emergency surgeries. Thereafter, one additional patient in group P discontinued the allocated intervention because of severe hypotension during anesthesia induction. Excluding 1 patient who deviated from the protocol, thirty-nine patients were finally analyzed in this study (group R, n = 20; group P, n = 19).

Patient demographics revealed no significant differences in background characteristics between the 2 groups (Table 1). The numbers of patients treated with antipsychotics, anxiolytics, hypnotics, antidementia drugs, and antiparkinsonian drugs were not significantly different. There were also no significant differences in intraoperative variables, including the duration of surgery and anesthesia, and the number of patients requiring fentanyl administration (Table 2). There were no cases where neurological evaluations using MEP monitoring were unavailable.

**Table 1:** Patient characteristics.

	Propofol (n = 19) 81	Remimazolam (n = 20) 81	*P* value
Age (yr), median [IQR]	81 [79–82]	80 [79–83]	>.99
Sex (Male/Female)	13/6	10/10	.33
Height (cm), median [IQR]	156.9 [151.2–162]	154.7 [147–158.9]	.59
Weight (kg), median [IQR]	58.5 [52.5–65.6]	56.8 [50.0–68.7]	.85
Body mass index, median [IQR]	22.9 [21.7–26.1]	23.6 [21.2–27.6]	.75
ASA class (2/3)	15/4	18/2	.41
Preoperative comorbidities (n)			
Cranial nerve dysfunction	1	0	.49
Cerebral infarction	3	0	.11
Renal dysfunction	5	7	.74
Liver dysfunction	1	1	>.99
Preoperative medications (n)			.30
Antipsychotics	2	2	
Anxiolytics and Hypnotics	1	5	
Antidementia drugs	0	1	
Anti-parkinsonian drugs	1	0	

IQR = interquartile range.

**Table 2:** Intraoperative variables.

	Propofol (n = 19)	Remimazolam (n = 20)	*P* value
Type of surgery (n)			
laminoplasty and/or laminectomy (cervical/lumber)	18 (3/15)	16 (1/15)	
resection of cervical spine tumors	0	2	
Posterior spinal fusion	1	1	
Other	0	1	
Duration of surgery (min), median [IQR]	111 [89–171]	105.5 [79–159.5]	.40
Duration of anesthesia (min), median [IQR]	184 [138–239]	147 [125–217]	.35
The rate of remifentanil administration (µg/kg/min)	0.23 [0.23–0.26]	0.24 [0.23–0.27]	.77
The average rate of propofol administration (mg/kg/h)	3.99		
The average rate of remimazolam administration (µg/kg/min)		0.93	
Administration of fentanyl (n)	3 (15.8%)	2 (10.0%)	.65
Administration of acetaminophen (n)	19 (100%)	20 (100%)	1.00
Administration of flurbiprofen (n)	13 (68.4%)	15 (75.0%)	.73
Administration of antiemetics (n)	0% (0%)	1 (5.0%)	>.99

IQR = interquartile range.

The emergence time (time to extubation) was significantly shorter in group R than in group P (4 minutes vs 8 minutes, *P <* .001; Table 3). The distribution of extubation times is shown in Table 4. In group R, the extubation times were < 5 minutes in 15 patients and 5 to 10 minutes in 5 patients. In group P, the extubation times were < 5 minutes in 3 patients, 5 to 10 minutes in 3 patients, 10 to 15 minutes in 10 patients, and > 15 minutes in 3 patients.

**Table 3:** Emergence variables.

	Propofol (n = 19)	Remimazolam (n = 20)	*P* value
Time to eyes opening (min)	7 [3,11]	2 [1,3]	.0001
Time to obeying commands (min)	7 [4,9]	2 [2,3]	<.0001
Time to extubation (min)	8 [6,12]	4 [3,4.75]	<.0001
Time to achieving a WFTS of 12 (min)	10 [7,15]	5 [3.25,5.75]	.0002

Values are expressed as median [interquartile range].

WFTS = white fast-track score.

**Table 4:** Comparison of extubation times for propofol and remimazolam anesthesia.

	Propofol (n = 19)	Remimazolam (n = 20)
Under 5 min	3	15
5–10 min	10	5
10–15 min	3	0
Over 15 min	3	0

Values are expressed as the number of cases.

Secondary outcomes were also shorter in group R. The time to eye-opening, obeying commands, and achieving a WFTS of 12 were also significantly shorter in group R (*P <* .001 for all variables; Table 5). Six patients in group R and 2 in group P had a WFTS of 13; 5 in group R and one in group P received a 1-point subtraction for consciousness. One patient in group R had a score of 12 because of a 1-point subtraction in consciousness and circulation. There was no difference in the number of patients who underwent point subtraction for consciousness between the groups (*P =* .18; Table 5).

**Table 5:** White fast-track score before leaving the OR.

	Propofol (n = 19)		Remimazolam (n = 20)	
	n (%)	Subtracted parameter	n (%)	Subtracted parameter
Score 14	17 (89%)		13 (65%)	
Score 13	2 (11%)	consciousness (n = 1), SpO_2_ (n = 1)	6 (30%)	consciousness (n = 5), nausea (n = 1)
Score 12	0 (0%)		1 (5%)	consciousness and circulation

OR = operation room, SpO_2_ = oxygen saturation.

## 4. Discussion

This study showed that the time to extubation, eye opening, obeying commands, and achieving a WFTS score of 12 were all significantly shorter under remimazolam-based anesthesia with flumazenil than under propofol-based anesthesia in older patients who underwent spinal surgery. There were no prolonged extubation (defined as 15 minutes or longer)^[10]^ in the remimazolam group. Prolonged extubation was observed in 3 patients in the propofol group.

Extubation time is an important factor in emergence from general anesthesia. Prolonged extubation decreases OR efficiency, including case turnover and use of OR time, and increases variable OR costs^[11]^ and adverse events, such as delayed recovery of airway reflexes.^[2]^ Therefore, reduction of extubation time is crucial for OR staff.

The type of anesthetic used can affect the time of emergence. Remimazolam is a short-acting benzodiazepine that acts on gamma-aminobutyric acid (GABA)_A_ receptors. It is rapidly broken down by tissue esterases into an inactive metabolite and has a stable context-sensitive half-time of 7 to 8 minutes in various settings. Pharmacokinetic modeling showed that remimazolam has high clearance and a small volume of distribution,^[12]^ that is, prolonged infusions or higher doses are unlikely to result in accumulation. Propofol is a short-acting anesthetic. Owing to its high lipophilicity, it is rapidly distributed in the central nervous system and other tissues, and acts on GABA_A_ receptors, inducing rapid sedation. Thereafter, it is rapidly redistributed to the peripheral compartments and metabolized primarily in the liver, resulting in its rapid emergence after bolus dosing or termination of a brief infusion. In contrast, the context-sensitive half-time of propofol varies from approximately 5 minutes for a short infusion to < 40 min after an 8-hour infusion.^[3]^

Both anesthetics are short-acting; however, remimazolam is less accumulative. Notably, the GABAergic effects of remimazolam can be antagonized by flumazenil. This suggested that remimazolam can provide a more rapid emergence than propofol, which does not antagonize antidotes. However, remimazolam anesthesia takes longer to emerge than propofol anesthesia.^[13,14]^ A randomized Phase IIb/III trial in Japan^[13]^ reported that remimazolam anesthesia takes longer to extubate than propofol when flumazenil was not used. Shimamoto et al analyzed the cause of delayed emergence from general anesthesia with remimazolam and reported that lower doses of remimazolam should be considered for patients that are overweight, older, and have low plasma albumin levels.^[15]^ In this study, we focused on older patients > 75 years old, and most were extubated within 5 minutes in the remimazolam anesthesia group with the use of flumazenil. The results showed that remimazolam is suitable for anesthesia in older patients owing to rapid emergence.

The emergence status was assessed using the WFTS. We have no post-anesthesia care unit, and take patients from the OR directly to the less extensively monitored wards. Therefore, we used the white fast-track scoring system incorporating the evaluation of emesis and pain in the modified Aldrete scoring system. This is commonly used for discharge criteria from the post-anesthesia care unit to postsurgical wards, to measure the ability for discharge from the OR.^[16,17]^ In the remimazolam group, 5 of the 20 patients did not achieve full recovery of consciousness despite the administration of flumazenil (0.5 mg), whereas no significant difference was observed between the 2 groups regarding WFTS. In addition, no apparent adverse events, such as respiratory depression or further loss of consciousness, occurred after returning to the ward. Although several cases of caution against re-sedation after remimazolam administration have been reported,^[18,19]^ our study indicated that the use of flumazenil can provide a safe opportunity to improve recovery times compared to propofol.

In spinal surgery with MEP monitoring, the influence of benzodiazepines on MEP is considered similar to that of propofol. Although studies on remimazolam are few, they are unlikely to interfere with MEP monitoring.^[20,21]^ In our study, MEP monitoring was not affected in any case.

This study had several limitations. First, remimazolam and propofol were adjusted based on electroencephalogram monitoring; however, minimal doses of both anesthetics were not established. We did not excessively reduce the dose to avoid intraoperative arousal. Therefore, it is possible that both propofol and remimazolam could have been further reduced.

Second, flumazenil (0.5 mg) was administered simultaneously as the discontinuation of anesthetic administration in all patients; however, an adequate dose and timing for flumazenil administration have not yet been established. There have been reports that 0.2–0.4 mg flumazenil administration following remimazolam anesthesia in older patients undergoing transcatheter aortic valve implantation did not cause re-sedation, and that 0.5 mg flumazenil administered immediately after remimazolam anesthesia did not cause any adverse events. In this study, we set up a 0.5 mg single-dose flumazenil protocol based on these reports.^[22–24]^ Third, the study could not be blinded to anesthesiologists; therefore, although unlikely, a performance bias may exist. Overall, We anesthesiologists are still inexperienced in the use of remimazolam. It may be possible that with more experience, the required dosage of this drug will become lesser. The appropriate dosage of flumazenil also needs to be considered. We believe it is necessary to reexamine this issue after several years of clinical use.

In conclusion, we investigated the state of emergence from remimazolam-based anesthesia in the older patients > 75 years old who underwent spinal surgery. This study showed that remimazolam-based anesthesia with flumazenil resulted in a significantly faster emergence than propofol-based anesthesia.

## Acknowledgments

We also would like to thank Editage (www.editage.jp) for English language editing.

## Author contributions

**Conceptualization:** Takashi Kondo.

**Data curation:** Ryuji Nakamura.

**Formal analysis:** Takashi Kondo, Satoshi Kamiya.

**Funding acquisition:** Takashi Kondo.

**Investigation:** Yukari Toyota, Kyoko Oshita, Toshiaki Haraki, Soshi Narasaki, Kenshiro Kido.

**Methodology:** Takashi Kondo.

**Project administration:** Takashi Kondo.

**Resources:** Yukari Toyota.

**Software:** Takashi Kondo.

**Supervision:** Noboru Saeki, Yasuo M. Tsutsumi.

**Validation:** Yukari Toyota.

**Visualization:** Yukari Toyota.

**Writing – original draft:** Yukari Toyota.

**Writing – review & editing:** Takashi Kondo, Yousuke T. Horikawa.

## Supplementary Material


