# Supplementary material for: Remimazolam-based anesthesia with flumazenil allows faster emergence than propofol-based anesthesia in older patients undergoing spinal surgery: A randomized controlled trial
Source: Medicine (Baltimore). 2023 Nov 17;102(46):e36081. doi: 10.1097/MD.0000000000036081 (PMC10659722; doi:10.1097/MD.0000000000036081)
Supplement: Supplementary file 1 [file medi-102-e36081-s001.docx]

| Supplementary Table S1. The White fast-track scoring system | |  |
| --- | --- | --- |
|  |  |  |
|  |  | Score |
| Consciousness | Awake and oriented | 2 |
|  | Arousable with minimal stimulation | 1 |
|  | Responsive to only tactile stimulation | 0 |
| Activity | Able to move all extremities | 2 |
|  | Some weakness in movement of extremities | 1 |
|  | Unable to voluntarily move extremities | 0 |
| Circulation | MAP ±15% of pre anesthesic level | 2 |
|  | MAP ±15-30% of pre anesthesic level | 1 |
|  | MAP >30% of pre anesthesic level | 0 |
| Breathing | Able to breathe deeply | 2 |
|  | Tachypneic with cough | 1 |
|  | Dyspnea with weak cough | 0 |
| SpO_2_ | SpO_2_ > 90% in room air | 2 |
|  | SpO_2_ > 90% on supplemental O_2_ | 1 |
|  | SpO_2_ < 90% on supplemental O_2_ | 0 |
| Pain | No pain or mild discomfort | 2 |
|  | Moderate to severe pain requires IV analgesics | 1 |
|  | Persistent and severe pain | 0 |
| Nausea and vomiting | None or mild nausea without vomiting | 2 |
|  | Transient nausea | 1 |
|  | Persistent moderate or severe nausea and active vomitimg | 0 |
|  |  |  |
| SpO_2_; oxygen saturation, MAP; mean arterial pressure, IV; intravenous, | |  |
|  |  |  |
